# (5-*n*-Hexyl-2-hydroxymethyl-1,3-dioxan-2-yl)methanol

**DOI:** 10.1107/S1600536810041401

**Published:** 2010-10-23

**Authors:** Min Zhang, Xian-You Yuan, Seik Weng Ng

**Affiliations:** a Department of Biology and Chemistry, Hunan University of Science and Engineering, Yongzhou Hunan 425100, People’s Republic of China; b Department of Chemistry, University of Malaya, 50603 Kuala Lumpur, Malaysia

## Abstract

In the title compound, C_12_H_24_O_4_, the dioxane ring adopts a chair conformation; the *n*-hexyl chain, which occupies an equatorial position, has an extended zigzag conformation. In the crystal, mol­ecules are connected by O—H⋯O hydrogen-bonds into a zigzag chain running along the *b* axis, giving rise to a herringbone pattern.

## Related literature

For a related structure, see: Luo *et al.* (2008 ▶).
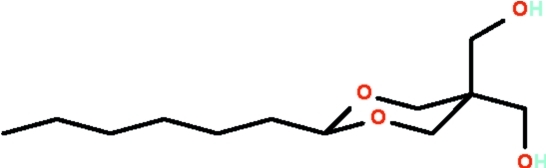


## Experimental

### 

#### Crystal data

C_12_H_24_O_4_
*M* *_r_* = 232.31
Monoclinic, 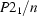

*a* = 13.6602 (10) Å
*b* = 5.9370 (5) Å
*c* = 16.4268 (12) Å
β = 97.737 (1)°
*V* = 1320.10 (18) Å^3^
*Z* = 4
Mo *K*α radiation
μ = 0.09 mm^−1^
*T* = 173 K
0.40 × 0.25 × 0.20 mm

#### Data collection

Bruker SMART APEX diffractometer
7438 measured reflections
2862 independent reflections
1755 reflections with *I* > 2σ(*I*)
*R*_int_ = 0.041

#### Refinement

*R*[*F* ^2^ > 2σ(*F* ^2^)] = 0.056
*wR*(*F* ^2^) = 0.173
*S* = 1.06
2862 reflections
154 parameters
2 restraints
H atoms treated by a mixture of independent and constrained refinement
Δρ_max_ = 0.32 e Å^−3^
Δρ_min_ = −0.24 e Å^−3^

### 

Data collection: *SMART* (Bruker, 2003 ▶); cell refinement: *SAINT* (Bruker, 2003 ▶); data reduction: *SAINT*; program(s) used to solve structure: *SHELXS97* (Sheldrick, 2008 ▶); program(s) used to refine structure: *SHELXL97* (Sheldrick, 2008 ▶); molecular graphics: *X-SEED* (Barbour, 2001 ▶); software used to prepare material for publication: *publCIF* (Westrip, 2010 ▶).

## Supplementary Material

Crystal structure: contains datablocks global, I. DOI: 10.1107/S1600536810041401/bt5377sup1.cif

Structure factors: contains datablocks I. DOI: 10.1107/S1600536810041401/bt5377Isup2.hkl

Additional supplementary materials:  crystallographic information; 3D view; checkCIF report

## Figures and Tables

**Table 1:** Hydrogen-bond geometry (Å, °)

*D*—H⋯*A*	*D*—H	H⋯*A*	*D*⋯*A*	*D*—H⋯*A*
O3—H3⋯O4^i^	0.84 (1)	1.86 (1)	2.664 (2)	162 (3)
O4—H4⋯O3^ii^	0.85 (1)	1.81 (1)	2.630 (2)	164 (3)

Symmetry codes: (i) 
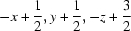
; (ii) 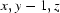.

**Fig. 1.**
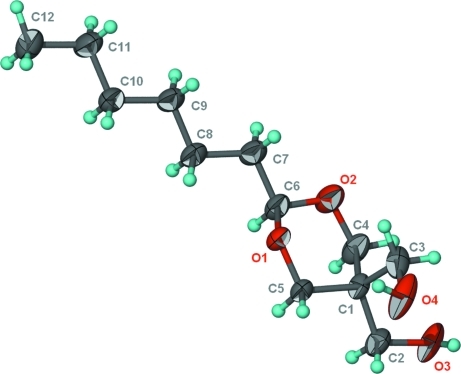
Anisotropic displacement ellipsoid plot (Barbour, 2001) of C12H24O4 at the 70% probability level; hydrogen atoms are shown as spheres of arbitrary radius.

**Fig. 2.**
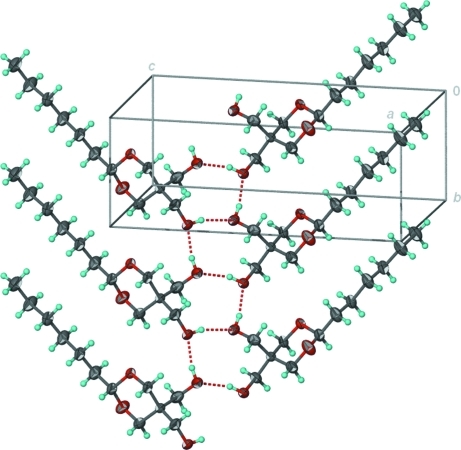
Hydrogen-bonded ribbon structure.

## supplementary crystallographic information

### Comment

A previous study reported the crystal structure of 5,5-bis(hydroxylmethyl)-2-phenylmethyl-1,3-dioxane, which was synthesized by the condensation of 2,2-bis(hydroxymethyl)-1,3-propanediol and an aromatic aldehyde (benzaldehyde) (Luo *et al.*, 2008). A variation of the synthesis with an aliphatic aldehyde under similar reaction conditions yielded a 1,3-dixoxane having the hydroxyl groups connected to another atom of the chair-shaped ring. In the molecule of the C_12_H_24_O_4 _(Scheme I, Fig. 1), the *n*-hexyl chain, which occupies an equatorial position, has an extended zigzag conformation. The hydroxy unit of one molecule is a hydrogen-bond donor to the hydroxy unit of an adjacent molecule so that the two O–H···O hydrogen bonds give rise to a herring-bone ribbon that runs along the *b*-axis of the monoclinic unit cell (Fig. 2).

### Experimental

2,2-Bis(hydroxymethyl)-1,3-propanediol (13.0 g, 96 mmol) and *N*,*N*-dimethylformamide (100 ml) were heated until the 2,2-bis(hydroxymethyl)-1,3-propanediol dissolved completely. *n*-Heptanal (10.1 g, 89 mmol) and *p*-toluenesulfonic acid monohydrate (1 g, 5 mmol) were added. The solution was heated 363–373 K 5 h. The solution was cooled and ethyl acetate (100 ml) was added to dissolve the residue after DMF was removed by evaporation. The solution was washed successively with water and 5% sodium bicarbonate (50 ml); the solution was dried over sodium sulfate. The solvent was evaporated to give a solid that was recrystallized from ethyl acetate to yield 16.5 g (70%) of colorless crystals.

### Refinement

Carbon-bound H-atoms were placed in calculated positions (C—H 0.95–0.99 Å) and were included in the refinement in the riding model approximation, with *U*_iso_(H) set to 1.2–1.5*U*_eq_(C).

The hydroxy H-atoms were located in a difference Fourier map, and were refined isotropically with a distance restraint of O–H 0.84±0.01 Å.

### Crystal data

**Table:**

C_12_H_24_O_4_	*F*(000) = 512
*M**_r_* = 232.31	*D*_x_ = 1.169 Mg m^−^^3^
Monoclinic, *P*2_1_/*n*	Mo *K*α radiation, λ = 0.71073 Å
Hall symbol: -P 2yn	Cell parameters from 2106 reflections
*a* = 13.6602 (10) Å	θ = 2.5–27.0°
*b* = 5.9370 (5) Å	µ = 0.09 mm^−^^1^
*c* = 16.4268 (12) Å	*T* = 173 K
β = 97.737 (1)°	Prism, colorless
*V* = 1320.10 (18) Å^3^	0.40 × 0.25 × 0.20 mm
*Z* = 4	

### Data collection

**Table:**

Bruker SMART APEX diffractometer	1755 reflections with *I* > 2σ(*I*)
Radiation source: fine-focus sealed tube	*R*_int_ = 0.041
graphite	θ_max_ = 27.1°, θ_min_ = 1.8°
ω scans	*h* = −17→17
7438 measured reflections	*k* = −7→7
2862 independent reflections	*l* = −20→14

### Refinement

**Table:**

Refinement on *F*^2^	Primary atom site location: structure-invariant direct methods
Least-squares matrix: full	Secondary atom site location: difference Fourier map
*R*[*F*^2^ > 2σ(*F*^2^)] = 0.056	Hydrogen site location: inferred from neighbouring sites
*wR*(*F*^2^) = 0.173	H atoms treated by a mixture of independent and constrained refinement
*S* = 1.06	*w* = 1/[σ^2^(*F*_o_^2^) + (0.0932*P*)^2^] where *P* = (*F*_o_^2^ + 2*F*_c_^2^)/3
2862 reflections	(Δ/σ)_max_ = 0.001
154 parameters	Δρ_max_ = 0.32 e Å^−^^3^
2 restraints	Δρ_min_ = −0.24 e Å^−^^3^

### Fractional atomic coordinates and isotropic or equivalent isotropic displacement parameters (Å^2^)

**Table:**

	*x*	*y*	*z*	*U*_iso_*/*U*_eq_	
O1	0.21236 (8)	1.1104 (2)	0.46544 (7)	0.0274 (3)	
O2	0.08583 (9)	1.3743 (2)	0.45699 (9)	0.0449 (4)	
O3	0.25706 (13)	1.7500 (2)	0.65335 (8)	0.0449 (4)	
H3	0.2562 (18)	1.699 (4)	0.7006 (8)	0.063 (8)*	
O4	0.24968 (15)	1.1821 (2)	0.68721 (8)	0.0551 (5)	
H4	0.257 (2)	1.051 (2)	0.6681 (16)	0.083 (9)*	
C1	0.22572 (13)	1.4192 (3)	0.56451 (10)	0.0259 (4)	
C2	0.29973 (15)	1.5895 (3)	0.60499 (12)	0.0358 (5)	
H2A	0.3529	1.5084	0.6402	0.043*	
H2B	0.3302	1.6693	0.5618	0.043*	
C3	0.17790 (16)	1.2926 (3)	0.62952 (12)	0.0390 (5)	
H3A	0.1398	1.3998	0.6590	0.047*	
H3B	0.1313	1.1794	0.6024	0.047*	
C4	0.14640 (15)	1.5360 (3)	0.50544 (12)	0.0387 (5)	
H4A	0.1046	1.6292	0.5369	0.046*	
H4B	0.1780	1.6370	0.4687	0.046*	
C5	0.27918 (13)	1.2561 (3)	0.51385 (11)	0.0275 (4)	
H5A	0.3173	1.3429	0.4774	0.033*	
H5B	0.3264	1.1648	0.5512	0.033*	
C6	0.14157 (13)	1.2334 (3)	0.41238 (11)	0.0321 (5)	
H6	0.1756	1.3257	0.3737	0.039*	
C7	0.07331 (14)	1.0673 (4)	0.36474 (12)	0.0391 (5)	
H7A	0.0179	1.1508	0.3333	0.047*	
H7B	0.0452	0.9678	0.4040	0.047*	
C8	0.12197 (14)	0.9224 (3)	0.30569 (12)	0.0342 (5)	
H8A	0.1780	0.8402	0.3368	0.041*	
H8B	0.1489	1.0211	0.2655	0.041*	
C9	0.05174 (14)	0.7536 (4)	0.25968 (13)	0.0393 (5)	
H9A	0.0248	0.6562	0.3002	0.047*	
H9B	−0.0043	0.8368	0.2290	0.047*	
C10	0.09718 (14)	0.6053 (4)	0.20005 (11)	0.0355 (5)	
H10A	0.1277	0.7026	0.1615	0.043*	
H10B	0.1505	0.5148	0.2311	0.043*	
C11	0.02531 (15)	0.4480 (4)	0.15108 (14)	0.0457 (6)	
H11A	−0.0268	0.5389	0.1186	0.055*	
H11B	−0.0069	0.3545	0.1897	0.055*	
C12	0.07088 (17)	0.2939 (4)	0.09337 (13)	0.0473 (6)	
H12A	0.1047	0.3843	0.0558	0.071*	
H12B	0.0189	0.2039	0.0617	0.071*	
H12C	0.1185	0.1936	0.1252	0.071*	

### Atomic displacement parameters (Å^2^)

**Table:**

	*U*^11^	*U*^22^	*U*^33^	*U*^12^	*U*^13^	*U*^23^
O1	0.0304 (7)	0.0258 (7)	0.0243 (6)	0.0012 (5)	−0.0030 (5)	−0.0047 (5)
O2	0.0375 (8)	0.0450 (9)	0.0482 (9)	0.0139 (7)	−0.0091 (7)	−0.0197 (7)
O3	0.0890 (12)	0.0223 (7)	0.0231 (7)	−0.0018 (7)	0.0063 (8)	−0.0032 (6)
O4	0.1172 (14)	0.0244 (8)	0.0241 (7)	−0.0002 (9)	0.0109 (8)	0.0009 (6)
C1	0.0359 (10)	0.0209 (9)	0.0211 (9)	−0.0008 (7)	0.0041 (7)	−0.0013 (7)
C2	0.0487 (12)	0.0298 (11)	0.0290 (10)	−0.0094 (9)	0.0054 (9)	−0.0058 (8)
C3	0.0559 (13)	0.0301 (11)	0.0349 (11)	−0.0091 (9)	0.0200 (10)	−0.0060 (8)
C4	0.0477 (12)	0.0305 (11)	0.0352 (11)	0.0093 (9)	−0.0046 (9)	−0.0078 (8)
C5	0.0278 (9)	0.0316 (10)	0.0221 (9)	−0.0014 (7)	−0.0002 (7)	−0.0045 (7)
C6	0.0344 (10)	0.0338 (11)	0.0259 (10)	0.0041 (8)	−0.0038 (8)	−0.0051 (8)
C7	0.0301 (10)	0.0468 (13)	0.0373 (11)	0.0054 (9)	−0.0064 (9)	−0.0119 (9)
C8	0.0335 (10)	0.0400 (12)	0.0269 (9)	−0.0021 (9)	−0.0034 (8)	−0.0051 (8)
C9	0.0288 (10)	0.0498 (13)	0.0364 (11)	0.0018 (9)	−0.0059 (9)	−0.0127 (9)
C10	0.0338 (10)	0.0437 (12)	0.0282 (10)	−0.0045 (9)	0.0014 (8)	−0.0039 (9)
C11	0.0353 (11)	0.0528 (14)	0.0464 (12)	−0.0015 (10)	−0.0035 (9)	−0.0196 (10)
C12	0.0541 (13)	0.0490 (14)	0.0388 (12)	−0.0057 (11)	0.0059 (11)	−0.0131 (10)

### Geometric parameters (Å, °)

**Table:**

O1—C6	1.415 (2)	C6—C7	1.503 (3)
O1—C5	1.421 (2)	C6—H6	1.0000
O2—C6	1.403 (2)	C7—C8	1.516 (3)
O2—C4	1.436 (2)	C7—H7A	0.9900
O3—C2	1.415 (2)	C7—H7B	0.9900
O3—H3	0.84 (1)	C8—C9	1.517 (3)
O4—C3	1.428 (3)	C8—H8A	0.9900
O4—H4	0.85 (1)	C8—H8B	0.9900
C1—C2	1.519 (2)	C9—C10	1.511 (3)
C1—C4	1.521 (3)	C9—H9A	0.9900
C1—C3	1.523 (2)	C9—H9B	0.9900
C1—C5	1.525 (2)	C10—C11	1.507 (3)
C2—H2A	0.9900	C10—H10A	0.9900
C2—H2B	0.9900	C10—H10B	0.9900
C3—H3A	0.9900	C11—C12	1.510 (3)
C3—H3B	0.9900	C11—H11A	0.9900
C4—H4A	0.9900	C11—H11B	0.9900
C4—H4B	0.9900	C12—H12A	0.9800
C5—H5A	0.9900	C12—H12B	0.9800
C5—H5B	0.9900	C12—H12C	0.9800
			
C6—O1—C5	111.39 (13)	O1—C6—H6	109.7
C6—O2—C4	112.06 (13)	C7—C6—H6	109.7
C2—O3—H3	109.7 (18)	C6—C7—C8	114.28 (15)
C3—O4—H4	106.1 (19)	C6—C7—H7A	108.7
C2—C1—C4	110.53 (16)	C8—C7—H7A	108.7
C2—C1—C3	110.16 (14)	C6—C7—H7B	108.7
C4—C1—C3	109.65 (15)	C8—C7—H7B	108.7
C2—C1—C5	108.80 (14)	H7A—C7—H7B	107.6
C4—C1—C5	107.08 (14)	C7—C8—C9	113.05 (16)
C3—C1—C5	110.57 (15)	C7—C8—H8A	109.0
O3—C2—C1	113.19 (15)	C9—C8—H8A	109.0
O3—C2—H2A	108.9	C7—C8—H8B	109.0
C1—C2—H2A	108.9	C9—C8—H8B	109.0
O3—C2—H2B	108.9	H8A—C8—H8B	107.8
C1—C2—H2B	108.9	C10—C9—C8	114.86 (16)
H2A—C2—H2B	107.8	C10—C9—H9A	108.6
O4—C3—C1	111.78 (16)	C8—C9—H9A	108.6
O4—C3—H3A	109.3	C10—C9—H9B	108.6
C1—C3—H3A	109.3	C8—C9—H9B	108.6
O4—C3—H3B	109.3	H9A—C9—H9B	107.5
C1—C3—H3B	109.3	C11—C10—C9	114.35 (16)
H3A—C3—H3B	107.9	C11—C10—H10A	108.7
O2—C4—C1	110.89 (15)	C9—C10—H10A	108.7
O2—C4—H4A	109.5	C11—C10—H10B	108.7
C1—C4—H4A	109.5	C9—C10—H10B	108.7
O2—C4—H4B	109.5	H10A—C10—H10B	107.6
C1—C4—H4B	109.5	C10—C11—C12	114.61 (17)
H4A—C4—H4B	108.0	C10—C11—H11A	108.6
O1—C5—C1	111.91 (13)	C12—C11—H11A	108.6
O1—C5—H5A	109.2	C10—C11—H11B	108.6
C1—C5—H5A	109.2	C12—C11—H11B	108.6
O1—C5—H5B	109.2	H11A—C11—H11B	107.6
C1—C5—H5B	109.2	C11—C12—H12A	109.5
H5A—C5—H5B	107.9	C11—C12—H12B	109.5
O2—C6—O1	111.11 (14)	H12A—C12—H12B	109.5
O2—C6—C7	108.74 (14)	C11—C12—H12C	109.5
O1—C6—C7	107.86 (15)	H12A—C12—H12C	109.5
O2—C6—H6	109.7	H12B—C12—H12C	109.5
			
C4—C1—C2—O3	62.40 (19)	C4—C1—C5—O1	−52.67 (19)
C3—C1—C2—O3	−58.9 (2)	C3—C1—C5—O1	66.74 (19)
C5—C1—C2—O3	179.70 (15)	C4—O2—C6—O1	60.4 (2)
C2—C1—C3—O4	−57.4 (2)	C4—O2—C6—C7	179.00 (16)
C4—C1—C3—O4	−179.27 (15)	C5—O1—C6—O2	−60.02 (18)
C5—C1—C3—O4	62.89 (19)	C5—O1—C6—C7	−179.11 (14)
C6—O2—C4—C1	−57.7 (2)	O2—C6—C7—C8	173.00 (17)
C2—C1—C4—O2	170.43 (14)	O1—C6—C7—C8	−66.4 (2)
C3—C1—C4—O2	−67.93 (19)	C6—C7—C8—C9	178.94 (17)
C5—C1—C4—O2	52.08 (19)	C7—C8—C9—C10	180.00 (18)
C6—O1—C5—C1	57.40 (18)	C8—C9—C10—C11	−176.53 (18)
C2—C1—C5—O1	−172.15 (14)	C9—C10—C11—C12	−178.08 (19)

### Hydrogen-bond geometry (Å, °)

**Table:**

*D*—H···*A*	*D*—H	H···*A*	*D*···*A*	*D*—H···*A*
O3—H3···O4^i^	0.84 (1)	1.86 (1)	2.664 (2)	162 (3)
O4—H4···O3^ii^	0.85 (1)	1.81 (1)	2.630 (2)	164 (3)

Symmetry codes: (i) −*x*+1/2, *y*+1/2, −*z*+3/2; (ii) *x*, *y*−1, *z*.
